# Construct validity and impact of mode of administration of the PedsQL™ among a pediatric injury population

**DOI:** 10.1186/s12955-014-0168-2

**Published:** 2014-11-30

**Authors:** Sami Kruse, Amy Schneeberg, Mariana Brussoni

**Affiliations:** British Columbia Injury Research & Prevention Unit, F508 – 4480 Oak Street, Vancouver, BC V6H 3V4 Canada; Child & Family Research Institute, Vancouver, BC Canada; Department of Pediatrics, University of British Columbia, Vancouver, BC Canada; School of Population & Public Health, University of British Columbia, Vancouver, BC Canada; British Columbia Children’s Hospital, Vancouver, BC Canada

**Keywords:** Child, Injury, Health related quality of life, Instrument, Measurement

## Abstract

**Background:**

The purpose of this study was to determine the construct validity of the PedsQL™ health related quality of life (HRQoL) instrument for use among injured children and to examine the impact of using different modes of administration, including paper and pencil, online and telephone.

**Methods:**

Two hundred thirty-three participants (aged 0 – 16) were recruited from hospital wards and the emergency department of a pediatric hospital in a large urban center in British Columbia, Canada. Data used to evaluate the construct validity of the PedsQL™ were collected from participants at the time of seeking injury treatment (baseline) to capture a retrospective measure of pre injury health, and one month post injury. Data used to compare different modes of administration (n = 44) were collected at baseline. To assess construct validity repeated measures analysis of variance (rANOVA) was used to determine whether the PedsQL™ tool was able to discriminate between patients pre and post injury while investigating possible interaction by category of length of stay in hospital. The impact of different modalities of administering the PedsQL™ on item responses was investigated using Bland-Altman plots.

**Results:**

rANOVA showed significant differences in PedsQL™ total score between baseline and one month post injury (*p < .001*), and differences in mean total score at one month post injury by category of injury severity (*p < .001*). There was also significant interaction by category of injury severity for the change in PedsQL™ total score from baseline to one month (*p < .001*). Pearson’s correlations were highly significant across three modalities of survey administration: paper and pencil, computer and telephone administration (range: .92 to .97, *p < .001*). Bland-Altman plots showed strong consistency.

**Conclusion:**

The PedsQL™ instrument is able to discriminate between pre and post injury HRQoL, as well as HRQoL post injury for injuries of varying severity. These findings are an indication that this instrument has good construct validity for the purpose of evaluating HRQoL of injured children. Data collected via paper-pencil, online and telephone administration were highly consistent. This is important as depending on the setting, clinical or research, different modalities of completing this instrument may be more appropriate.

## Background

According to the World Health Organization, injuries include lesions from acute exposure to energy exceeding the threshold of physiological tolerance and/or impairment of function resulting from a lack of one or more vital elements (i.e. air, water, warmth), as in strangulation, drowning or freezing [1]. Injuries range from being minor scrapes and bruises to severe trauma requiring hospitalization and sometimes may result in death. Unintentional injuries are said to occur when there is no intent to harm. The leading mechanisms include traffic collisions, drowning, poisoning, falls and burns [1].

Unintentional injuries are the leading cause of mortality in Canada among individuals 1-34 years of age. In 2009, 663 children age 1-14 years and 2,096 individuals age 15 to 24 years died as the result of an unintentional injury [2]. Unintentional injuries are also the leading cause of hospitalization in Canada among children 10-14 years and the second largest cause of hospitalization among children 5-9 and 15-19 years [3]. While injuries among young people are commonplace, the long-term sequelae are not well understood. Outcome data are needed to gain understanding of the breadth of impact on children and youth. Despite the major burden that injuries represent, studies investigating health related quality of life (HRQoL) after traumatic injury are scarce, particularly for children and youth, and for those who were not admitted to hospital as a result of their injuries [4]. There is only one study on HRQoL among injured children and youth that includes children treated and released from the emergency department and children admitted to hospital [5]. This study of children aged 5 to 14 years found that admission to hospital and length of stay, particularly of >3 days, were negatively associated with children’s functioning at 2.5 months, five months and nine months post injury.

Measuring HRQoL in pediatric settings has a number of benefits, including the ability to facilitate communication between patient and care provider, as well as to assist with clinical decision making [6]. Furthermore, capturing not only the physical, but also the psychological and social effects of injury can help direct health services and health research on this topic and provide practitioners with a sense of the broader impact of an injury on a child’s wellbeing that is not readily understood based on typical hospital data.

Numerous instruments have been developed to collect information on children’s HRQoL. Disease specific questionnaires can provide valuable information when used in a particular setting [7]. However, these instruments are not available for all types of disease, necessitating the use of generic instruments designed for multiple populations with varying conditions. Generic instruments should be psychometrically validated for use among specific populations. Construct validity is one of the characteristics of a questionnaire that deserves careful consideration. Construct validity is the extent to which what is measured by the tool corresponds to the theoretical concepts of the phenomenon under study [8]. A HRQoL tool with good construct validity for injured populations would be able to identify differences between pre and post injury HRQoL as well as differences in impact on HRQoL for severe relative to less severe injuries. Capturing these differences in HRQoL is important as this allows for a better understanding of the impact of an injury on a child’s health and wellbeing. In order to assess construct validity, a pre injury measure of HRQoL is required. The literature suggests that capturing a retrospective measure of pre injury HRQoL is superior to using population norms [9,10].

Also of interest is the influence that mode of questionnaire administration might have on results. Paper and pencil, computer and telephone administration have been recognized as commonly used methods to collect HRQoL data [11,12]. Online administration of questionnaires is gaining increasing prominence in health research and can be an effective and efficient way of collecting data on health status and health-related outcomes [13]. Some researchers suggest that computer versions of health questionnaires are becoming especially popular among young audiences accustomed to using computers and the internet [4]. Telephone administration remains relevant as it provides researchers with the opportunity to explain instrument items that may be unclear to the participant [12]. Moreover, telephone administration can be particularly valuable in injury research as it provides an alternative method of response to those who cannot write or type due to injury. Research has been inconclusive regarding whether participant responses vary across different modes of questionnaire administration, with some finding significant differences [14], and others finding few differences [15]. To maximize flexibility in administration and utility of measures, it is important to understand the impact of using different modes of administration, including paper and pencil, online and telephone.

The PedsQL™ 4.0 Generic Core [16] and the PedsQL™ Infant Scales [17] are two generic instruments that are commonly used to measure HRQoL in children and youth aged 0 to 18 years. However, psychometric evaluation of these tools in a population of injured children is limited [18]. The objectives of this study were twofold. First, we sought to examine the construct validity of the PedsQL™ to evaluate its appropriateness for use in a population of injured children. Second, we aimed to examine the influence of using different modes of administration, including paper and pencil, online and telephone, on results obtained from a population of injured children and youth. The *a priori* hypothesis was that if the construct validity of the PedsQL was strong: (1) there would be a difference in pre injury and post injury HRQoL total scores, and (2) HRQoL total score one month post injury would vary by hospital length of stay. (3) We further hypothesized that the use of different modes of survey administration would not impact the validity of the data collected.

## Methods

### Study sample

Injured children and youth and their parent or primary caregiver were recruited in the emergency department and hospital in-patient units of a pediatric hospital in a large urban centre in British Columbia, Canada. Participants were eligible to participate if the child was 0 to 16 years of age and had a primary injury diagnosis for which he or she was seeking treatment, the parent or primary caregiver and child (aged five years and up) were able to speak English, and the family resided in the province of British Columbia, Canada. Potential participants had the study explained to them and were given written information about the study by a research assistant. Parents or caregivers agreeing to participate provided written consent, and children aged seven and over gave written assent for participation in the study. After consent was obtained, while still in the hospital, each participant was given a questionnaire package that included the PedsQL™ instrument, a series of questions relating to the nature of the injury, including the body part injured, type of injury (break, sprain, burn etc.) and how the injury occurred, as well as questions about patient demographics, including the child’s age, sex, and parent’s income. To assess construct validity participants were mailed a follow-up package one month post injury that included the PedsQL™ instrument. Ethics approval for all study procedures was obtained from the hospital and university ethics board and Public Health Agency of Canada Research Ethics Board.

### Instruments

The PedsQL™ 4.0 Generic Core and the PedsQL™ Infant Scales were developed to assess HRQoL in children, ages 2 to 18 years, and 0 to 24 months respectively. The standard version of these instruments was used in this study. The PedsQL™ 4.0 Generic Core is a 23 item scale and includes four subscales of functioning: physical, emotional, social and school [16]. The PedsQL™ Infant Scales are instruments composed of 36 items for infants 1-12 months and 45 items for toddlers 13-24 months each with five subscales: physical functioning, physical symptoms, emotional functioning, social functioning and cognitive functioning [17]. Both instruments also allow for the calculation of a psychosocial scale; the mean score of all items in the emotional, social, and school functioning subscales. These instruments use a five point Likert response scale ranging from “never” to “almost always” to assess the extent to which different items have affected the child in the previous month. For both measures, individual item scores were obtained by reverse scoring items and linearly transforming them to a scale of 0 to 100, with higher scores indicating better HRQoL. Total scores were obtained by adding the sum of items and dividing them by the number of items answered.

Children aged 8 to 16 years provided self-report PedsQL™ data, and children 5 to 7 years completed the questionnaire with assistance from a parent or caregiver. Self-report continues to be recognized as the standard for measuring quality of life; however, parental proxy has been identified as a viable collection method for children who are not able to answer questions themselves [19]. In this study, parents completed the PedsQL™ instrument for children of all ages, providing us with two sources of HRQoL data. For the analyses herein, child data were used when available and parent proxy data were used for those under the age of five and in cases where children were unable to complete instruments due to injury.

### Data collection

The PedsQL™ was administered in the hospital at the time of recruitment in order to capture a retrospective measure of pre injury health (baseline) as well as one month post injury to assess the HRQoL changes in children after an injury. Recruitment for this study started February 2011 and continued until November 2011. The follow-up questionnaires were mailed to participants. Participants were given a CDN$2 voucher to a local merchant for each questionnaire they completed. The primary diagnosis and other injury data were abstracted from the hospital records. If required, participants could take the baseline questionnaire home and mail it in.

For assessment of the impact of different modes of questionnaire administration, participants recruited from hospital in-patient units were invited to be part of a sub-sample to complete the baseline PedsQL™ scale three times over the span of one day, each time via a different mode of administration – paper and pencil, online and telephone. Mode of administration order was randomized. Recent literature reporting on the validity of questionnaire administration methods shows time between administering questionnaires ranging from five minutes [20] to several weeks [21]. Because the quality of life in an injured patient is subject to rapid changes after an injury event, collecting data at three time points over one day was chosen to ensure changes in the participant’s health status were not likely to affect responses. By using the in-patient population, rather than the emergency department, for this portion of the study we had access to participants throughout the day for survey administration at different time points. Upon completion, each participant was asked their most and least preferred mode of administration and provided with an honorarium of CDN$30.

### Statistical analyses

All data analyses were conducted using SPSS version 20.0 software. Descriptive statistics were used to describe the demographic characteristics of the sample.

### Construct validity

Construct validity was examined by exploring the relationship between injury severity and HRQoL at two time points: at baseline to capture a retrospective measure of pre injury health and one month post injury. Previous research on pediatric injuries has used length of hospitalization as a surrogate measure for injury severity, with longer hospital stays representing more serious injuries [6]. This proxy measure has been validated and found suitable for use, particularly in pediatric populations where the risks of co-morbidity and medical fragility are low [22]. As such, to examine the relationship between injury severity and HRQoL, we used length of stay as a proxy for injury severity and divided into three categories: not admitted, one to three days in hospital, and four or more days in hospital. The Bonferroni correction [23] was applied to the post-hoc analysis in order to account for multiple comparisons made when assessing the level of differences between categories of length of stay.

Repeated measures analysis of variance (rANOVA) was used to determine whether the PedsQL tool was able to discriminate between patients pre and post injury, while examining for interactions with categories of length of stay in hospital. Post-hoc analyses were performed to examine differences across groups of injury severity. Mean differences and 95% confidence intervals between scores were also calculated to test for both statistical and clinical differences. Differences of 4.4 for child report, and 4.5 for parent proxy are sufficiently clinically relevant to mandate a change in the patients’ clinical management [24].

### Impact of mode of administration

The impact of different modalities of administering the PedsQL on item responses was investigated by comparing modalities using the Bland-Altman method [25]. Pearson’s correlation coefficients were calculated to measure the strength of association between two variables at a time (i.e., paper and pencil compared with online; online compared with telephone; and telephone compared with paper and pencil). Pearson’s correlation provides a measure of linear association between paired observations. Values close to zero indicate very weak correlation and values >0.95 indicate quasi-perfect linear correlation [26]. The differences between the total PedsQL scores were then computed and difference scores compared two methods at a time. The average mean difference in scores for each pair of modalities were calculated and plotted. The level of agreement of modalities was assessed by observing the scatter of the differences and evaluating the number of points beyond two standard deviations of the mean.

## Results

### Study sample

No significant differences in age, gender, and injury severity were observed between those who completed both time points versus those who completed only one.

To investigate the impact of mode of survey administration, we invited those who were initially recruited from in-patient units (n = 60) to participate and 45 participants (75%) agreed. One participant was not able to complete the process due to challenges with timing and therefore 44 participants participated in testing multiple modalities of administration. All but one participant also completed the one month follow-up and were included in the analysis for the construct validity. Demographic details of the sample for both study arms are provided in Table 1.

**Table 1:** **Participant demographics**

		**Construct validity**	**Reliability of modalities**
		**N = 233 (%)**	**N = 44 (%)**
**Respondent**	Self-report	140 (60)	11 (25)
	Parent proxy report	93 (40)	33 (75)
**Treatment**	Emergency department	178 (76)	0
	Admitted 1-3 days	31 (13)	21 (48)
	Admitted 4+ days	24 (10)	22 (50)
	Unknown	0	1 (2)
**Age of injured child (years)**	0-2	31 (13)	6 14)
	2-4	50 (21)	9 (20)
	5-7	40 (17)	5 (11)
	8-12	62 (27)	11 (25)
	13-16	50 (21)	14 (32)
**Sex of injured child**	Boy	144 (62)	26 (59)
	Girl	89 (38)	18 (41)
**Injury type**	Head injury	36 (15)	3 (7)
	Upper extremities fracture	48 (21)	5 (11)
	Lower extremities fracture	18 (8)	6 (14)
	Dislocation, sprain and strain	23 (10)	3 (7)
	Minor external	36 (15)	1 (2)
	Internal organ	2 (1)	1 (2)
	Facial	3 (1)	0
	Other*	67 (29)	25 (57)
**Family income**	Above national median	170 (73)	20 (45)
	Below national median	51 (22)	13 (30)
	Not specified	12 (5)	11 (25)

*Includes open wounds, burns, injury to nerves, dental injury, poisoning, and asphyxia.

### Construct validity

**Figure 1:**
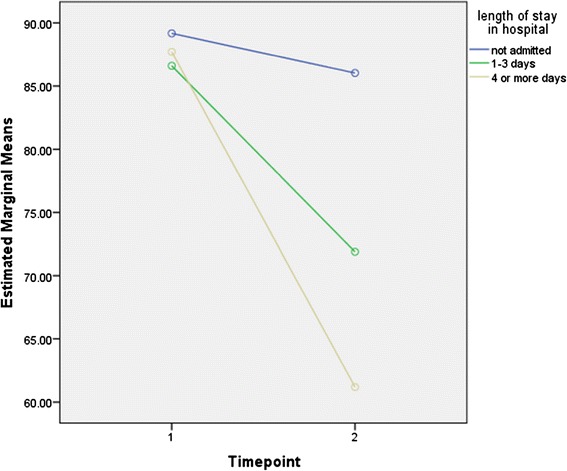
**Estimated Marginal Means of PedsQL scores at baseline and 1 month post injury.**

HRQoL scores from each category of length of stay were not significantly different at baseline, indicating comparable pre injury health status. The difference in total PedsQL™ mean scores at one month post injury relative to baseline was statistically significantly greater than zero based on 95% CI for all three categories of length of stay in hospital (Table 2), although only the two categories who had been hospitalized had clinically significant differences. The results of the rANOVA on total PedsQL scores showed that overall pre and post scores were statistically significantly different (F =21.51, *p* < .001). There were also significant differences in total PedsQL scores between categories of length of stay in hospital at one month (F =113.05, *p* < .001). Moreover, the relationship between pre and post injury scores was significantly different between categories of length of stay (F =31.13, *p* < .001), indicating that relationship between scores pre and post injury was modified by categories of length of stay. As expected, the greatest drop in HRQoL score pre and post injury was observed among children who spent four days or more in hospital: 26.52 (95% CI: 22.45, 30.59). In Figure 1 the relationship between pre injury and post injury HRQoL by injury severity is illustrated and the estimated marginal PedsQL means, both pre and post injury, are modeled.

**Table 2:** **PedsQL™ mean scores at baseline and one month post injury**

	**n**	**Baseline mean (95% CI)**	**One month follow-up mean (95% CI)**	**Mean difference (95% CI)**
Not admitted	178	89.17 (87.66, 90.68)	85.03 (84.03, 88.03)	3.14 (1.29, 5.00)
Admitted 1-3 days	31	86.60 (84.11, 89.08)	71.89 (68.78, 75.00)	14.71 (10.95, 18.47)
Admitted 4+ days	24	87.71 (84.74, 90.68)	61.19 (57.80, 84.74)	26.52 (22.45, 30.59)

Statistically and clinically significant differences were found when comparing those admitted for one to three and four days or more with those not admitted to the hospital (Table 3).

**Table 3:** **Differences in PedsQL™ mean score between categories of length of stay at one month post injury**

		**Mean difference (95% CI)**	**P-value**
Not admitted	Admitted 1-3 days	8.36 (3.35, 13.36)	<.001
Not admitted	Admitted 4+ days	13.15 (7.55, 18.74)	<.001
Admitted 1-3 days	Admitted 4+ days	4.79 (-2.20, 11.79)	.300

### Mode of administration

**Figure 2:**
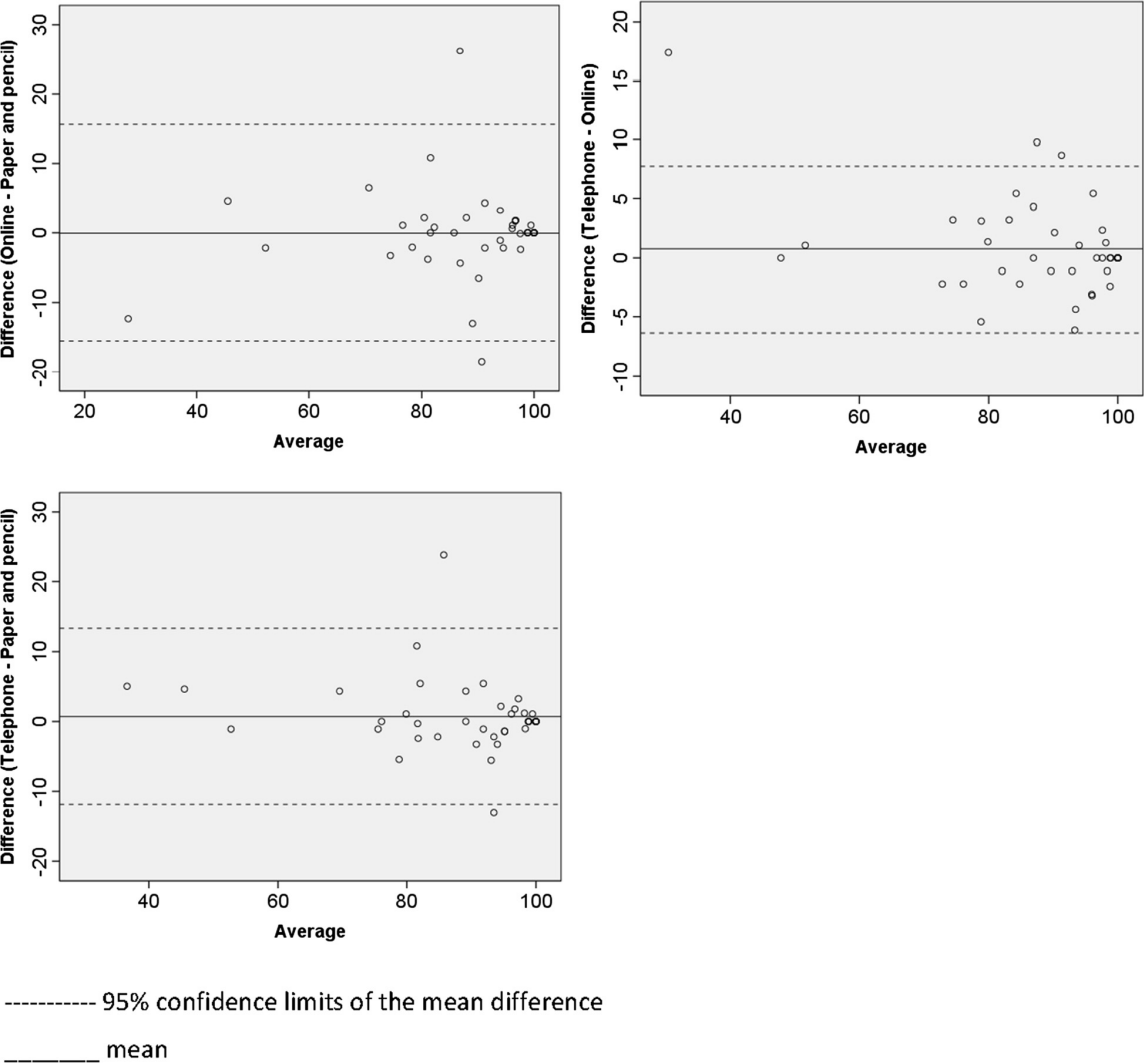
**Bland-Altman plots for Total PedsQL scores.**

Pearson’s correlations between total PedsQL scores across the three modalities were highly significant, ranging from 0.92 to 0.97 (*p* < .001). Overall, the Bland-Altman plots show considerable consistency across the different modalities of questionnaire administration (Figure 2). At most only three data points lay beyond two standard deviations of the mean when comparing the mean difference scores for each of the modalities. The majority of participants preferred internet based administration (54%), followed by paper and pencil (26%) and phone administration (20%).

## Discussion

The significant burden that injuries represent for children and youth highlights the importance of obtaining comprehensive outcome data to understand the scope of the injury and inform provision of clinical and rehabilitation services [27,28]. The purpose of this study was to assess the construct validity and impact of mode of administration of the PedsQL™ 4.0 Generic Core and the PedsQL™ Infant Scales among a pediatric injury population. Our findings support our hypotheses and show significant differences between the baseline retrospective pre injury PedsQL™ scores and those one month post injury suggesting that the PedsQL™ instruments are able to distinguish between groups of injured and non-injured children. Further, we found significant differences in PedsQL™ total scores one month post injury between categories of length of stay in hospital indicating that the PedsQL™ is able to discriminate between minor and more severe injuries. Finally, our results also demonstrate that survey responses did not vary across different modes of survey administration.

The instrument has been identified as a suitable measure for outcome assessment of children after trauma [18,29], however psychometric evaluation and confirmation of utility within a pediatric injury population remains limited [18]. To our knowledge, only one other study exists that examines the validity of the PedsQL™ for assessing outcomes in pediatric injury patients [30]. Our findings are consistent with this previous research, which showed the PedsQL™ to be a suitable instrument for use among injured children. In 2012, Stevens et al. recruited in an emergency department setting and collected PedsQL™ data two weeks post injury [30]. Our study builds on these authors’ finding as it includes children recruited in both the emergency department as well as hospital in-patient units and collects PedsQL™ data one month post injury. Thus, our methods are consistent with the standard PedsQL™ instrument design that asks respondents to consider the previous month in their answers [31], and also collects data from both the hospital emergency department and in-patient units, potentially reaching children experiencing a broader spectrum of injury severity.

Our findings provide evidence that data collected via paper-pencil, online and telephone administration of the PedsQL™ instruments are highly consistent and highly correlated. Thus, alternative methods of administration can be considered that are most appropriate for different trauma sites and patients, without compromising data quality. Most participants identified online administration as their preferred form of instrument completion. This might prove the most efficacious form of survey administration as it has reduced costs [32] and the potential to provide instantaneous information on a patient’s health status [33]. Online administration has the potential to introduce bias with regard to socioeconomic factors such as age, income and education due to differential access to the internet among different population groups [32]. However, Canadian research indicates widespread internet access and use across the population [34].

The significant findings in this study add novel information to existing literature on assessing HRQoL among injured children. This is only the second study to look at the HRQoL of injured children who have been treated and released from hospital, and the first to investigate the psychometric properties of the PedsQL™ in this population. Further, we utilized a very inclusive study population with a wide age range as well as varied type and severity of injury, making our results generalizable to the larger pediatric injury population. Nonetheless, important study limitations should be noted. Despite oversampling, the small number of participants with longer length of stays in hospital limited statistical power and the number of investigations possible. In the multiple modality reliability assessment portion of the study, participants completed all three questionnaire modalities in one day. While this may have increased recall of responses to questionnaire items, in the interest of limiting measurement differences due to actual HRQoL changes, we chose to undertake data collection within this timeframe. Furthermore, the hospital environment contains considerable distractions, such as interruptions or noise from medical personnel, other patients or visitors, which may have made it difficult for participants to concentrate and could have impacted their sense of confidentiality. Although some participants took the baseline questionnaire home to complete, we feel that this did not impact the validity of our measure of pre injury HRQoL. In their study evaluating the use of retrospective reporting to assess pre injury HRQoL, Watson et al. used the most immediate measure possible (average 4 days post injury, more than 15% of participants took the survey home from the hospital to complete) and concluded this retrospective measure was more appropriate than population norms, even for those who responded from home up to 1 week post injury [9]. Wilson et al took a retrospective measure of pre injury HRQoL on average 3.2 months post injury and concluded this measure is still better than population norms, although it may result in a slight bias towards over estimating HRQoL [10].

## Conclusion

In this study we sought to ensure that the PedsQL™ was suitable for use among a pediatric injury population and to test the comparability of multiple methods of administration to maximize ease of injury surveillance and standardized long-term follow-up of injured patients. Our findings confirm the PedsQL™ 4.0 Generic Core and the PedsQL™ Infant Scales are appropriate instruments for collecting HRQoL data among injured children and that these data can be collected using paper-pencil, online or telephone modes of administration without compromising data quality.
